# Hidradenitis suppurativa: epidemiology, diagnosis, molecular pathogenesis and therapy

**DOI:** 10.1186/s43556-026-00495-4

**Published:** 2026-07-07

**Authors:** Maria Grazia Lolli, Luca Sanna, Roberta Belli, Anna Dattolo, Luca Fania, Laura Colonna, Paolo Marchetti, Cristina Albanesi, Laura Mercurio, Stefania Madonna

**Affiliations:** 1 Laboratory of Experimental Immunology, Fondazione Luigi Maria Monti, Istituto Dermopatico Dell’Immacolata-Istituto Di Ricovero E Cura a Carattere Scientifico IDI-IRCCS, Via Monti di Creta, 104, Rome, 00167 Italy; 2 Dermatology Unit, Istituto Dermopatico Dell’Immacolata IDI-IRCCS, Rome, 00167 Italy; 3 Department of Oncology, Istituto Dermopatico Dell’Immacolata IDI-IRCCS, Rome, 00167 Italy

**Keywords:** Hidradenitis suppurativa, Epidemiology, Molecular pathogenesis, Obesity, ECM remodeling, Fibrosis, Therapies, Adipokines, Immunity

## Abstract

Hidradenitis suppurativa (HS) is a chronic, debilitating inflammatory skin disease with a global prevalence ranging from 0.3–1%, characterized by recurrent nodules, abscesses, and sinus tracts that culminate in irreversible tissue fibrosis. Despite its impact on patient quality of life, the transition from acute inflammation to chronic tissue remodeling remains poorly understood. Comorbidities frequently accompany skin alterations. HS pathogenesis is complex and involves innate immune cells, Th1/Th17-cell responses, and B-cell mechanisms, which lead to irreversible skin damage with tunnel formation. This review synthesizes current knowledge on HS epidemiology, clinical features and molecular mechanisms driving its development and progression, with a focus on the pathogenic role of adipose tissue, and specifically of adipokines, on inflammation and tissue destruction. We delineate how systemic metabolic dysfunction, often associated with obesity, synergizes with local inflammation to alter the balance between MMPs and their tissue inhibitors TIMPs, thus contributing to a perpetuating circle of inflammation and fibrosis. Furthermore, we explore the impact of conventional and targeted therapies on the interruption of broad or specific inflammatory and fibrotic pathways to reduce disease activity, and restore skin homeostasis. Vice versa, we illustrate how the dysregulated metabolic-inflammatory axes influence the therapeutic outcomes emphasizing how the adipose tissue microenvironment modulates the response to conventional and targeted therapies. Finally, we discuss how targeting the interplay between metabolic health and tissue destruction represents a promising frontier for preventing the permanent skin damage in advanced disease, offering the potential for more tailored and effective intervention strategies.

## Introduction

Hidradenitis suppurativa (HS) is a chronic, debilitating inflammatory skin disease characterized by recurrent nodules, abscesses, and sinus tracts that culminate in irreversible tissue fibrosis [1, 2]. The epidemiology of HS displays substantial geographic and demographic heterogeneity. Given an estimated global prevalence of around 1%, HS also has considerable socioeconomic relevance to society [3–5]. Indeed, HS has a considerable effect on the personal and professional lives of those affected, most of whom are young or middle-aged adults. In Europe and North America, the disease predominantly afflicts women, with incidence peaking between the second and fourth decades of life.

In recent years, important advancements have been made in HS research. The aetiology of HS is complex and multifactorial, involving genetic predisposition, environmental triggers like smoking, and significant systemic comorbidities, including metabolic, cardiovascular, musculoskeletal, gastrointestinal, and mood disorders. Obesity is the most frequent comorbid condition, affecting up to 75% of patients and acting as a systemic inflammatory amplifier [6]. Beyond standard cytokines, adipose tissue secretes specialized signaling proteins called adipokines, which regulate systemic inflammation, metabolism, and immune responses, thus contributing to HS development and progression [7, 8].

Disease severity evolves from recurrent nodules in acute forms to diffuse involvement with interconnected tunnels and extensive scarring in chronic phases. The poorly understood transition from acute inflammation to chronic and irreversible tissue remodeling remains a primary scientific gap. In the care of patients with HS, the fundamental gap is characterized by a significant delay in diagnosis and a subsequent "therapeutic lag" in receiving effective treatments. Patients often face a diagnostic delay of about 7–10 years from the onset of symptoms to an accurate diagnosis [9]. To this matter, robust genetic and proteomic biomarkers that could facilitate early differential diagnosis actually lack. Furthermore, biologics and advanced targeted therapies are frequently introduced only after years of less effective systemic treatments, allowing the disease to progress toward irreversible tissue damage. Of note, there is a 40–50% non-response rate to current biologics, which is largely driven by a lack of understanding regarding how complex genetic and metabolic profiles influence individual clinical outcomes [1]. In light of the emerging therapeutic strategies, further efforts should be addressed to the identification of biomarkers useful to predict the clinical outcomes in HS patients.

In this review, we provide a comprehensive overview of HS, encompassing epidemiology, comorbidities, diagnostic strategies, molecular pathogenesis, and current standards of care. Building upon this foundation, we synthesize emerging insights into genetic predisposition, as well as into cellular and molecular players involved in HS pathogenesis, illustrating the complex immune-stromal framework to explain HS manifestation and progression. By linking metabolic dysfunction, and specifically obesity-related adipokines, to local inflammation and tissue destruction strongly sustained by metalloproteinases (MMPs) and their inhibitors (Tissue Inhibitors of Metalloproteinases—TIMPs), we aim to outline translational strategies for precision-guided therapies in HS and to highlight future directions focused at integrating the influence of the "gut-skin axis" and metabolic factors on HS pathogenesis and the optimal response to drugs.

## Epidemiology and clinical burden of HS

### Geographical heterogeneity of HS

Epidemiology of HS displays substantial geographic and demographic heterogeneity. Global prevalence estimates typically range from 0.3–1%, though reports vary widely -from 0.001% to over 4%- largely due to methodological and diagnostic differences across studies [9].

A recent meta-analysis estimated a pooled global prevalence of approximately 0.99% [10]. In some parts of the world, HS predominantly afflicts women to men by a ratio of 3:1 and most often presents in young adults, with incidence peaking in the second to fourth decades of life [11]. In Europe and North America, HS is consistently female-predominant (≈2–3:1), whereas several East Asian studies report a male predominance, highlighting possible genetic or environmental modulators [12]. In the United States, population-based claims analyses indicate higher prevalence among Black individuals compared with White or Asian groups [13]. Regionally, epidemiologic data are strongest in Europe and North America, while reliable population-level studies remain limited in Africa, Latin America, and parts of Asia [14]. This disparity in HS prevalence may be attributed to socio-economic determinants that influence lifestyle factors, such as diet, smoking, wound care, and weight management [15].

In addition, geographic heterogeneity in HS prevalence likely reflects population-specific genetic architectures and differential exposure to environmental inflammatory triggers. These observations highlight the importance of integrating epidemiologic data with molecular profiling to identify population-specific pathogenic pathways and therapeutic targets.

### Diagnosis, clinical features and disease severity assessment

HS is a chronic, recurrent, folliculo-centric inflammatory disease characterized by painful, deep-seated nodules, abscesses, draining tunnels (sinus tracts), and progressive scarring in intertriginous sites.

HS diagnosis is clinical and is based on the on the modified Dessau criteria, which include the observation of the typical lesions, their characteristic distribution, and the history of recurrence. Specifically, the primary lesions appear as single tender inflammatory nodules (1–2 cm in diameter), often mistaken for furuncles. They often begin as "blind boils" (meaning they lack a central pustular head) because the root inflammation sits deep within the hair follicle and dermis. These nodules can persist for weeks and develop into abscesses [16], accompanied by fluctuance, severe pain, and purulent drainage. The abscesses can evolve in elongated, purulent, fluctuant draining tracts (sinus tracts) beneath the skin, tombstone comedones, which manifest as multi-headed, open blackheads characteristic of chronic, damaged follicles, and progressively in hypertrophic scars in intertriginous sites, particularly the axillae, groin, perineal, and inframammary regions [17]. A second diagnostic criteria concerns lesions distribution, as HS has a strict predilection for intertriginous zones—areas where skin rubs against skin, rich in terminal hair follicles and apocrine sweat glands. Primary zones are axillae, inguinal folds (groin), perineum, perianal region, and buttocks. Inframammary and intermammary folds are highly common in women, while lesions can occasionally appear also on the neck, torso, or thighs.

Finally, the standard diagnostic threshold requires at least two recurrences within a 6-month period, or a continuous presence of lesions fluctuating in severity over months [18].

**Fig. 1:**
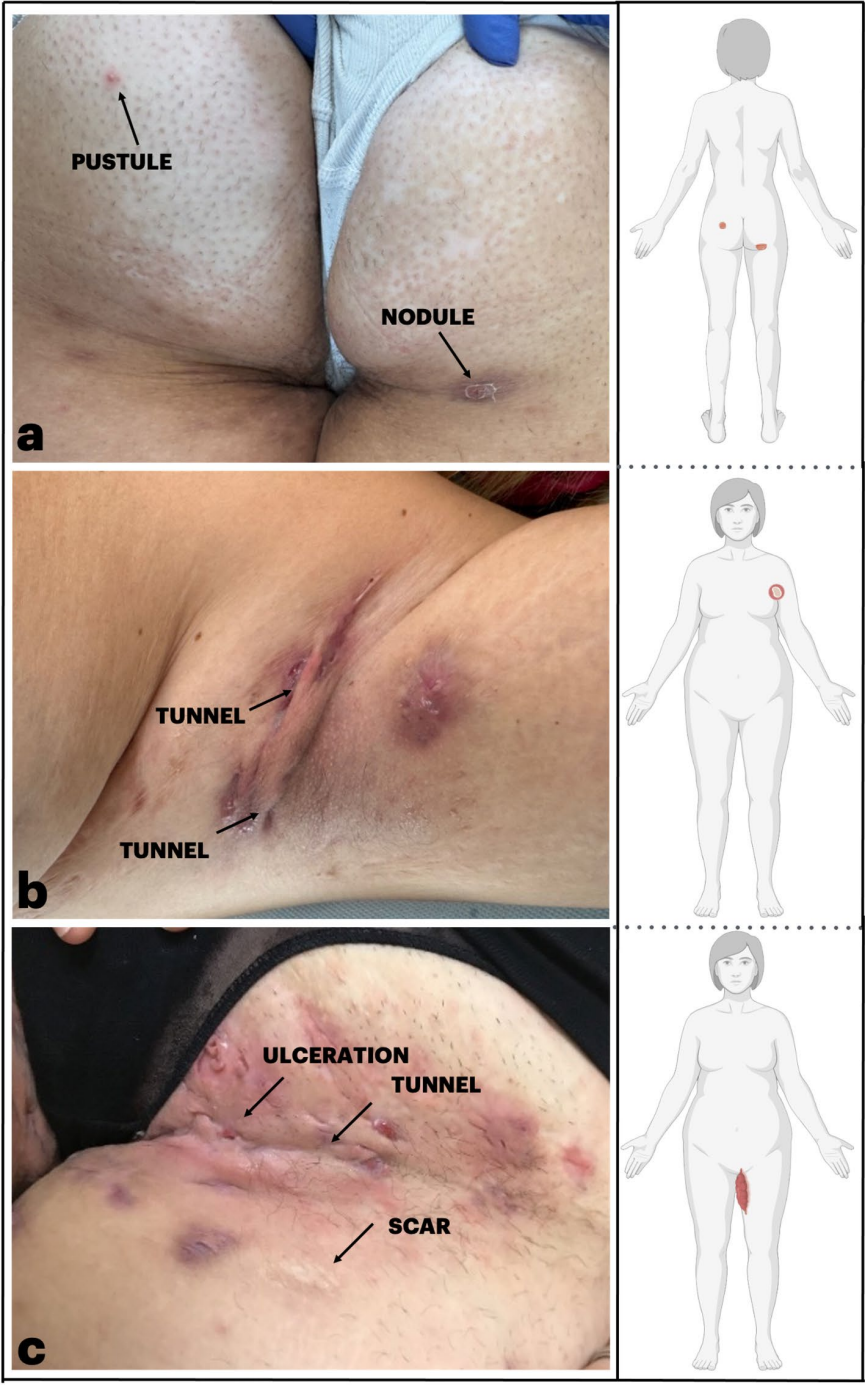
Representative clinical images illustrating Hurley disease severity stages in HS. The panel shows different types of Hurley Stages I, II and III of skin lesions in patients with HS. **a** Clinical presentation of a gluteal region in a female patient classified as Hurley stage I. The image shows solitary inflammatory nodule and pustule without sinus tract formation or scarring, localized to the gluteal region; **b** Hurley stage II disease in a female patient, characterized by two sinus tract formation localized in the axillary region.** c** Severe HS in a female patient classified as Hurley stage III. The image shows extensive sinus tract formation in the groin, with residual scarring and ulceration indicative of chronic and previous inflammatory activity

Once a diagnosis is confirmed, Hurley staging system (Hurley I-III) is typically used to classify the disease severity and the clinical course. Hurley I corresponds to recurrent nodules or abscesses without tunnels or scarring; Hurley II to widely separated recurrent abscesses with early sinus tracts and scarring; Hurley III to diffuse involvement with interconnected tunnels, extensive scarring, and chronic suppuration [18] (Fig. 1). As Hurley scoring does not allow a precise assessment of disease severity or improvement with drug treatment, there is a broad consensus to use the International Hidradenitis Suppurativa Severity Score System (IHS4), which is a validated score suitable for graded assessment of HS severity [19]. IHS4 is a sum of nodules (multiplied by one), abscesses (multiplied by two), and draining tunnels (multiplied by four), and it permits the classification of the disease severity as mild, moderate, and severe. The achievement of IHS4 reduction of 55% (IHS4-55), 75% (IHS4-75), and 90% (IHS4-90) is frequently used in clinical studies as endpoints [20].

However, while the Hurley Staging System remains the gold standard for clinical severity stratification, the field is increasingly integrating high-frequency ultrasound (HFUS) to identify subclinical tunnels and inflammatory activity that escape visual inspection [21].

HS has a significant impact on physical, mental, social, and professional domains. The chronic nature of the disease often leads to unpredictable flare-ups, causing a lot of distress. Several scales and indices have been developed to evaluate the effect of HS on Quality of Life (QoL). These tools, like the Dermatology Life Quality Index (DLQI) and the Hidradenitis Suppurativa Quality of Life Index (HiSQOL)**,** provide Patient-Reported Outcome Measures (PROMs) that help to assess the severity of the condition, track treatment progress, and measure the psychological and functional burden of disease.

From a translational standpoint, the molecular complexity of HS highlights the urgent need for robust biomarkers of disease activity, progression, and therapeutic response. Integration of molecular biomarkers with clinical severity scores such as IHS4 may enable precision medicine approaches, optimizing patient stratification and guiding early, targeted intervention.

### Risk factors and systemic comorbidities

**Fig. 2:**
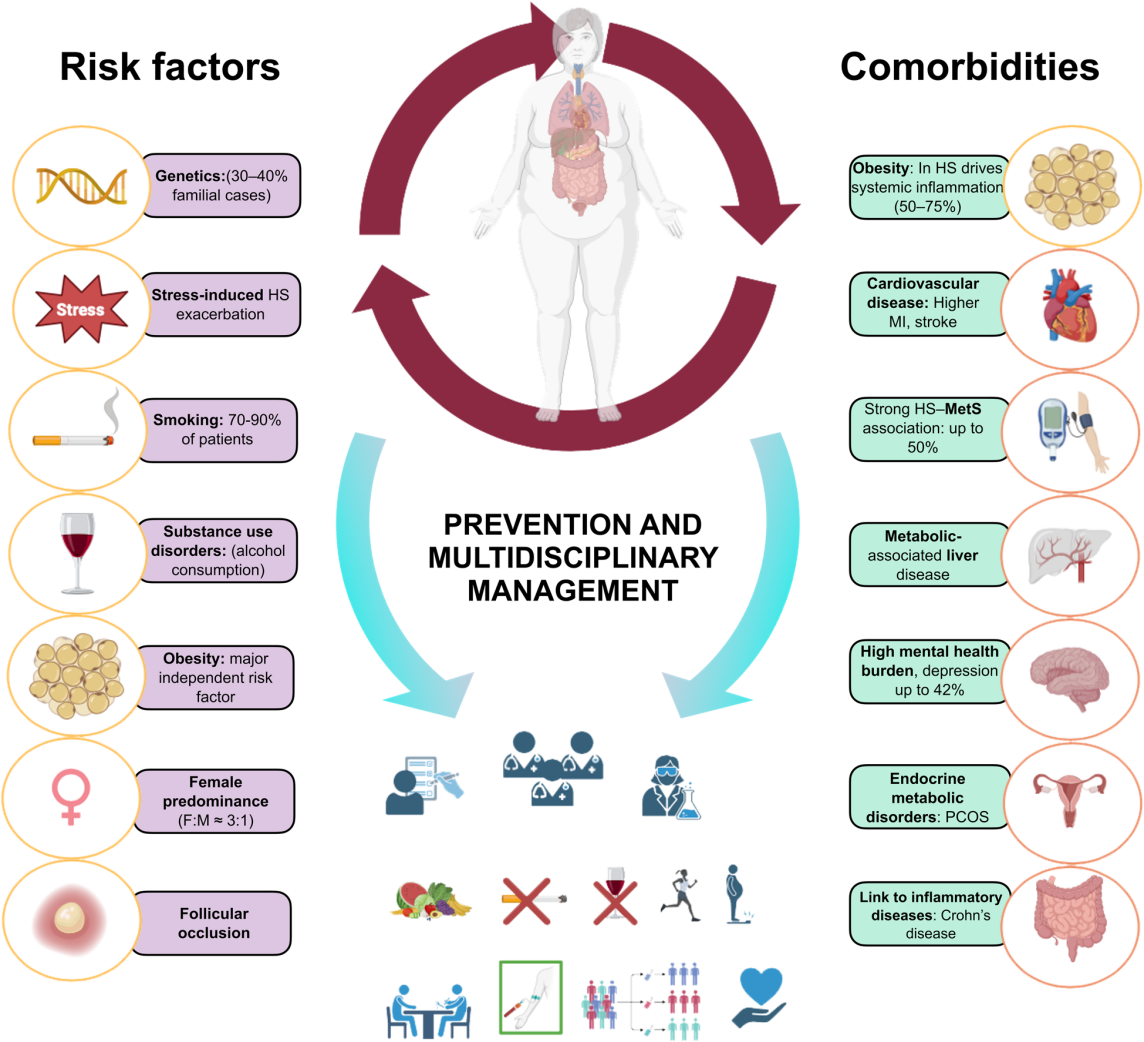
Risk factors and comorbidities associated with HS. Schematic representation of the risk factors and comorbidities associated with HS, with relative incidence percentages. HS is a systemic inflammatory disease with multifactorial etiology involving genetic predisposition, environmental triggers, and behavioral factors, with a higher prevalence in females. Obesity represents one of the major independent risk factors of HS and one of the most frequent comorbidities associated with the disease. In addition to obesity, the right panel of the scheme depicts the main comorbidities associated with HS. The Fig. highlights the importance of a multidisciplinary approach to the management of HS patients. Abbreviations: F, female; M, male; MI, myocardial infarction

HS is characterized by a complex and multifactorial etiology involving genetic predisposition, environmental triggers (i.e., stress), and behavioral factors (alcohol consumption, smoking), with a higher prevalence in women (ratio Female:Male 3:1) [22] (Fig. 2). Sex-specific differences are also evident in disease progression and comorbidity profiles, suggesting the role of sex-related hormones in HS pathology.

Androgens (like testosterone) are often the primary drivers in HS. They stimulate the sebaceous glands and hair follicles, leading to increased oil production and follicular plugging—the initial step in an HS lesion.

In women many report "flares" tied to the menstrual cycle (specifically the premenstrual phase when estrogen drops and progesterone or androgen influence shifts), suggesting that even cyclical hormonal fluctuations can trigger inflammation. In men HS often presents with more severe "atypical" involvements, such as back and nuchal (neck) involvement, potentially due to higher systemic androgen levels, while in women lesions are more frequently found in the inguinal, submammary, and axillary regions. Sex hormones also influence the metabolic and endocrine systems, leading to different "side-kick" conditions. Women are more likely to have Polycystic Ovary Syndrome (PCOS), central obesity, and thyroid issues. The insulin resistance associated with PCOS can further spike androgen levels, creating a feedback loop that worsens HS. Likely driven by a greater prevalence of tobacco use, men with HS show higher rates of cardiovascular disease and certain associated inflammatory conditions like squamous cell carcinoma [23]. Mechanical factors such as friction, mechanical pressure, and shear forces in intertriginous areas act as local triggers, promoting follicle rupture and subsequent spread of inflammation [24].

The most frequent comorbidity condition in HS is obesity, affecting up 50% to 75% of the HS patients and acting as a systemic inflammatory amplifier. It is considered an important independent factor in the development of HS and is associated with a higher prevalence of HS in children and adolescents [25]. Obesity exacerbates the inflammatory state and promotes follicular occlusion, while hyperlipidemia may amplify inflammation through oxidative stress and impaired immune resolution [4, 26] (Fig. 2). Indeed, the chronic low-grade inflammation associated with obesity can perpetuate the immune dysregulation characteristic of HS, by heightening Th17 signaling and increasing neutrophil recruitment [27]. In line with these observations, studies on bariatric surgery and significant weight loss (over 15% of body weight) have shown a direct correlation with reduced HS severity [15]. As a consequence of obesity, up to 50% of patients are affected by MetS, with insulin resistance up to of 77% regardless of disease duration, predisposing patients to type 2 diabetes mellitus and hypertension [28] (Fig. 2). Systemic inflammation inherent to HS can also critically alter lipid metabolism. This alteration promotes the hepatic overproduction of triglyceride-rich lipoproteins and hinders the clearance of LDL particles. Elevated pro-inflammatory cytokines like TNF-α and IL-6 further impair lipid regulation, contributing to dyslipidemia and atherosclerosis through cytokine-mediated endothelial dysfunction [29–31]. This compromised metabolic profile translates into a substantially increased risk of major adverse cardiovascular events, including myocardial infarction and ischemic stroke [32]. Recent evidence also identified a striking association with Metabolically Dysregulated-associated Steatotic liver disease (MASLD), which may affect up to 75% of patients [33]. HS also shares pathogenic pathways with immune-mediated diseases such as inflammatory bowel disease (IBD), particularly Crohn’s disease, and spondyloarthritis [34], reflecting common IL-23/Th17-driven mechanism [35] (Fig. 2). Other comorbidities include PCOS [36], sexual dysfunction, and ocular conditions such as scleritis and corneal ulcers [37–39]. Finally, chronic inflammation associated to HS exposes patients to an increased risk of cancer, particularly squamous cell carcinoma (SCC) and lymphomas [40] (Fig. 2).

Emerging evidence suggests that also psycho-neuro-endocrine pathways are significantly associated to HS pathophysiology [4, 41]. High rates of depression and anxiety are common (up to 42% of HS patients) at all levels of disease severity [42]. Other psychiatric disorders like schizophrenia, bipolar disorder, and disordered eating behaviors link also to HS. Of note, disordered eating further contributes to obesity, inflammation, and disease progression [43]. Thus, the comprehensive management of HS should move from a skin-centric model to a prevention and multidisciplinary strategies focused on reducing the overall inflammatory burden, in particular obesity [44] to avoid irreversible tissue damage (Fig. 2).

## Pathogenesis of HS: the complexity of molecular and cellular dysregulations

The pathogenesis of HS is a complex, multi-tiered, spatio-temporal process that bridges inherited genetic vulnerability with a highly destructive cellular cascade. This section explores how the disease evolves from upstream genetic and epigenetic predispositions to downstream, self-perpetuating tissue damage. First, we outline the genetic and epigenetic landscape that dictates the baseline reactivity and structural vulnerability of the cutaneous microenvironment. We then track how these molecular prompts orchestrate a dynamic immune-stromal transition, driving the cutaneous tissue through progressive clinical phases—from acute, reversible innate immune activation, through organized adaptive immunity and early dermal tunnel formation, to the irreversible, end-stage fibro-destructive loops that distort the skin architecture.

### Genetic architecture, inheritance and epigenetic rewiring: upstream drivers of cellular dysregulation

Multiple factors are known to contribute to HS onset. Indeed, while environmental and lifestyle factors alongside hormonal influences contribute to its pathogenesis, the strong familial disease clustering suggests also a significant genetic susceptibility. Specifically, 30 to 40% of patients report to have a positive family history of disease even though the genetic basis of HS is still unclear. Many evidences identified, in a minor subset of patients, pathogenic variants with autosomal dominant inheritance and incomplete penetrance in genes encoding for components of the Gamma-secretase complex (GSC), an intramembrane protease complex that in skin primarily processes Notch receptors [45]. Specifically, loss of function variants identified in Nicastrin (NCSTN), Presenilin Enhancer 2 (PSENEN2), Presenilin 1 (PSEN1), Aph-1A and Aph-1B Gamma-Secretase Subunits (APH1A and APH1B) genes, lead to haplo-insufficiency that ultimately results in defective Notch signaling [46–50]. Proper Notch pathway activation is a pivotal determinant for the regulation of skin homeostasis, keratinocytes differentiation, and the maintenance of the hair follicle, oil glands and skin appendages structure. Therefore, defective Notch pathway alters keratinocytes proliferation and differentiation program, compromises apocrine gland homoeostasis and switches the fate of outer root sheath hair follicle cells, converting it into keratin-enriched epidermal cysts, a typical phenotypic trait of HS [45, 51, 52]. Notch signaling also influences Treg development and function, so its deficiency further exacerbates the Th17/Treg imbalance present in HS [53]. Therefore, germline loss-of-function variants in the GSC subunits genes can directly trigger the clinical manifestation of HS by compromising Notch-mediated follicular integrity and immune homeostasis. However, only a very small fraction—approximately 5%—of familial HS cases follows Mendelian autosomal inheritance of monogenic dominant variants in GSC components genes. The vast majority of the disease's genetic architecture is non-Mendelian, driven by polygenic risk factors interacting with environmental triggers that remains largely uncharacterized [46, 48, 54].

Genome-Wide Association Studies (GWAS) identified common genetic variants, specifically Single Nucleotide Polymorphisms (SNPs), associated with HS susceptibility, located in loci of SOX9 and KLF5 genes [55, 56]. In skin, SOX9 is involved in the development and maintenance of hair follicles and sebaceous glands homeostasis, in fibrosis processes and in the formation of sinus tracts, through the control hair follicle stem cells, all processes that result alerted in HS [57, 58]. KLF5 instead regulates the skin barrier, tissue regeneration, and keratinocyte differentiation. Alterations in KLF5 functions can cause excessive keratin production within the follicular duct, leading to comedones formation and follicular occlusion, which are typical features in HS. It also modulates the local immune response, and its dysfunction contributes to the typical persistent inflammation of HS patients [59]. In sporadic HS cases, GWAS identified common SNPs in HLA-DR loci and in the pro-inflammatory genes TNF-α, IL-23R, IL-12RB1 involved in the regulation of innate and adaptive immune responses. This polygenic landscape could modulate the threshold for self-perpetuating chronic inflammation and follicular occlusion, thereby predisposing to the development of HS. Variants in HLA-DR region can lead to an aberrant presentation of commensal skin microbiota or follicular auto-antigens to CD4^+^ T-cells facilitating the persistent recruitment of Th17 and Th1 cells into the perifollicular space [55, 60, 61].

Another crucial aspect of non-Mendelian HS predisposition involves epigenetic alterations that could be inherited or acquired through environmental factors like smoking and obesity. Epigenetic alterations associated to HS include chromatin remodeling and aberrant DNA methylation of cytokine genes (like IL-17F and CXCL10), that can result in a persistent and self-amplifying pro-inflammatory state even in the absence of a primary genetic variants [62, 63].

Despite growing insights, defining the complete genetic landscape in HS pathogenesis, remains to be fully elucidated as a critical area of investigation to fully characterize the disease’s pathophysiology and advance targeted therapeutic strategies. However, it is plausible that these hereditary genetic variations and epigenetic modifications dictate the baseline reactivity of the cutaneous microenvironment by priming local cell populations, and instigating the complex cellular crosstalk explored in the following section.

### The immune-stromal landscape of HS: orchestrating the transition from innate activation to tertiary lymphoid structures and irreversible tissue alterations

The molecular mechanisms as well as cellular and immune contribution underlying HS pathogenesis, still remain incompletely defined, because of a severe lack of reliable preclinical models [64]. HS is a solely human disease and genetic mouse models, such as those with altered Notch signaling pathways, have shown multiple limitations, including the lack of altered immune responses and destructive tunnel-forming nature typically observed in human HS [64]. Therefore, human ex vivo models consisting of skin explants cultured in vitro have been developed to study HS pathogenesis, although they exhibit high donor variability often driven by the anatomical site from which they derive or intrinsic factors [64].

Lesional skin of HS patients is characterized by a heterogeneous inflammatory infiltrate, along with tissue damage, extracellular matrix (ECM) remodeling, and fibrosis [62, 65, 66]. The chronicization of initial inflammation and aberrant tissue repair, progressively disrupts skin cytoarchitecture and fibrosis [67].

**Fig. 3:**
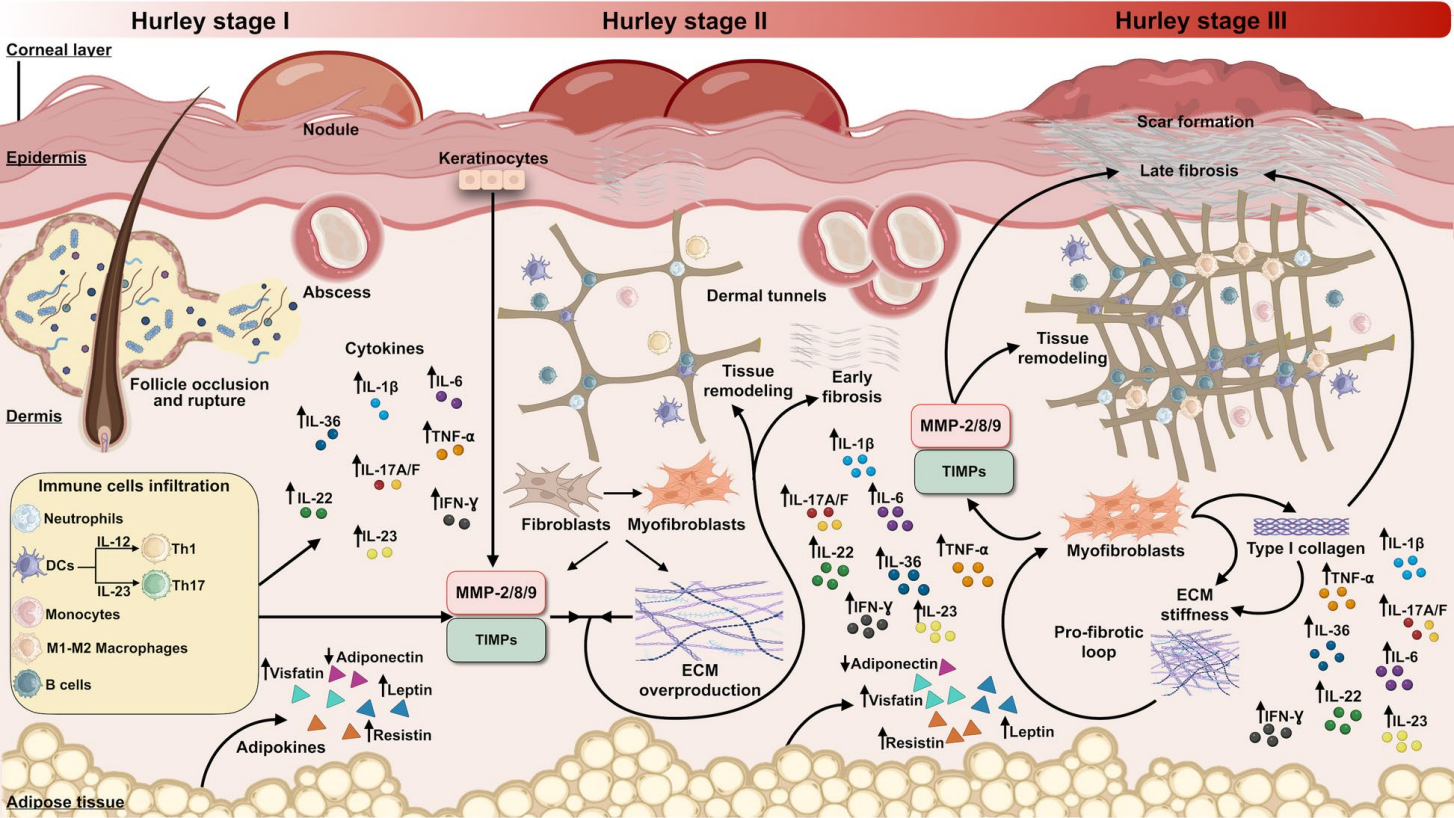
Molecular pathophysiology across Hurley stages of HS. In Hurley stage I, follicular occlusion of the pilo-sebaceous unit leads to rupture and dermal release of sebum, keratin, cellular debris, and bacteria, triggering an acute inflammatory response. The immune infiltrate primarily includes neutrophils, and DCs that lead the activation of Th1 and Th17 lymphocytes, monocytes, macrophages, and B cells. Key cytokines involved are IL-1β, TNF-α, IL-23, IL-17A/F, IL-6 and IL-36, IFN-γ, and IL-22. In parallel, the inflammatory milieu affects subcutaneous adipose tissue, promoting adipocyte release of pro-inflammatory adipokines as leptin, visfatin, and resistin, while decreasing anti-inflammatory adiponectin levels. Collectively, these events drive the formation of inflammatory nodules and abscesses. In Hurley stage II, the balance between MMP −2, −8, −9 and their tissue inhibitors TIMPs released by immune cells, keratinocytes, and fibroblasts activated by local inflammation, contributes to excessive ECM deposition, the increasing of ECM degradation biomarkers and tissue remodeling. This process favors the development of dermal tunnels enriched with immune infiltrates. Persistent inflammation, sustained by the continuous over-release of pro-inflammatory adipokines and cytokines, promotes early fibrotic changes and the onset of initial scarring typical of stage II. Hurley stage III is characterized by chronic, non-resolving inflammation driven by the persistent dysregulation of both pro-inflammatory cytokines and adipokines, together with defective wound healing. These mechanisms induce the transition of fibroblasts into activated myofibroblasts, which overproduce collagen type I and ECM components, leading to increased matrix stiffness and the establishment of a self-amplifying pro-fibrotic loop. The dysregulated activity of MMPs (−2, −8, −9) and TIMPs exacerbates tissue destruction, deepens dermal tunneling, and results in extensive fibrosis and scar formation typical of advanced Hurley stage III disease

The Hurley Stage I is the earliest phase of HS development, characterized by nodules and abscesses and it is dominated by acute inflammation where innate immune response drives the adaptive activation. It begins with the occlusion of the hair follicle, the consequent rapture of the pilosebaceous unit and the release of follicular contents, including keratin fragments, sebum, hair shaft remnants, microbial products, and cellular debris into the surrounding dermis resulting into inflammatory response and tissue damage (Fig. 3) [68]. These events act as a trigger that activates innate immune pathways through pattern-recognition receptors (PRRs) expressed by keratinocytes [69], macrophages [70], and dendritic cells [62, 71]. Keratinocytes and macrophages respond by secreting pro-inflammatory cytokines IL-1β, TNF-α, IL-6, IL-23, and IL-36 family members together with chemokines including CXCL1 and CXCL8, thereby orchestrating the rapid recruitment of neutrophils and monocytes to the site of follicular injury [72–74]. In this phase, macrophages shift toward pro-inflammatory CD68^+^ M1-phenotype [75] contributing to inflammation amplification that opposes wound healing. These cytokines promote T-helper CD4^+^ differentiation towards Th1 and Th17 subsets, significantly increased in HS lesions, which secrete IL-17A, IL-17F, IL-22, and IFN-γ cytokines [76, 77]. The activation of IL-17 signaling further induces the expression of CXCL1, CXCL8 and CCL20 in keratinocytes, thus sustaining leukocyte recruitment through self-reinforcing inflammatory feed-forward loops. In this phase, the action of immune infiltrate and keratinocytes contributes to ECM disruption and remodeling, by secreting MMPs, among which MMP-2, MMP-8 and MMP-9, and their modulators TIMPs. In this early stage of HS, subcutaneous adipose tissue also represents a key contributor of the disease pathogenic circuit. Indeed, adipose tissue acts as an endocrine immunological hub by secreting the pro-inflammatory adipokines leptin, visfatin and resistin with a concomitant decrease of the anti-inflammatory adipokine adiponectin (Fig. 3). These mediators sustain systemic and cutaneous immune activation, and induce the expression of MMPs and TIMPs, as well as of pro-fibrotic genes, thereby promoting inflammation, ECM remodeling and tissue fibrosis [78, 79]. However, in Hurley Stage I, tissue damage is still reversible and fibrosis is not yet present. This phase resembles the early inflammatory phase of wound healing, during which provisional matrix proteins as fibrin and fibronectin assemble into a scaffold stabilizing the injured site, providing a substrate for cellular migration and tissue healing [80].

The transitions from the acute phase of Hurley Stage I to a chronically self-sustaining inflammatory state, occurs with irreversible tissue damage when inflammatory signaling fails to resolve. This stage, called Hurley Stage II, is characterized by recurrent nodules and abscesses and the first signs of irreversible tissue alterations defined by the formation of early sinus tracts (dermal tunnels) and early fibrosis scarring [81–83] (Fig. 3). Immunologically, in Hurley Stage II, the persistent action of pro-inflammatory M1 CD68^+^ macrophages impedes wound healing and drive the prolonged chronic inflammation [72] in which CD163⁺ macrophages with M2-like features emerge, implicated in ECM deposition and fibrotic remodeling [76, 77]. Integrated transcriptomic and immunohistochemical analyses demonstrated the persistence of the Th1 and Th17-associated cytokines IL-23, IL-17A and IL-17F in this stage. IL-36 family cytokines further amplify both Th1 and Th17 responses, reinforcing pathogenic immune circuits [77]. Moreover, IL-17A and TNF-α act synergistically to drive a marked upregulation of the chemokines CCL20 and CXCL8 sustaining leukocyte recruitment [4, 84]. B-lymphocytes and plasma cells emerge as important contributors to disease chronicity. Histological analyses and single-cell and spatial transcriptomic profiling reveal tertiary lymphoid structures (TLSs) in lesional HS skin, characterized by organized interactions among T cells, B cells, antigen-presenting cells, and stromal elements underscoring the transition from predominantly innate to organized adaptive immune responses in Stage II disease [67, 68] (Fig. 3).

Persistent inflammation and dysregulated adipokines production, associated with the unperturbed action of MMPs and TIMPs acting on ECM, prevent the wound healing. In this phase, dermal activated fibroblasts play a central role in tissue remodeling and fibrosis. Single-cell RNA sequencing and spatial transcriptomic studies have recently identified distinct fibroblast subpopulations, including SFRP4⁺ and CXCL13⁺ [85], that modulate fibrosis [3]. CXCL13^+^ fibroblasts exhibit the most prominent expression of multiple MMPs, suggesting their direct contribution to tissue disruption and remodeling. Thus, MMPs activity in HS is not just a consequence of inflammation but is also tied to specific fibroblast phenotypes and dysregulated signaling pathways involving also subcutaneous adipose tissue within the HS skin microenvironment (Fig. 3). Activated fibroblasts also drive the upregulated deposition of ECM components in HS lesions, in part through the activated Hippo pathway, a pro-fibrotic driver in chronic HS [86]. Dermal fibroblasts progressively differentiate toward a myofibroblast phenotype [87], characterized by increased expression of contractile and ECM-associated genes such as ACTA2 and COL1A1 [88], that promote early sinus tracts formation and collagen accumulation. Thus, sinus tract formation and early fibrosis result as the outcome of the combined action of inflammation circuit, tissue disruption by MMPs and TIMPs with the simultaneous overproduction of ECM and pro-fibrotic components (Fig. 3).

The progression from Hurley Stage II to Stage III reflects the convergence of long-standing chronical inflammation, persistent dysregulations of fibroblasts and adipocytes and fibrosis spread. Hurley stage III, the most severe, is characterised by extensive irreversible tissue damages, advanced pervasive fibrosis, multiplication of sinus tracts and extension of scarring across the entire anatomical region [78] (Fig. 3). Hippo pathway dysregulation, detectable already in Stage II, is further amplified within expanded fibroblast populations, resulting in excessive collagen deposition and increased ECM stiffness that act on fibroblasts through mechano-transduction pathways, creating a "pro-fibrotic positive feedback loop" where the ECM mechanical properties enhance the fibrotic process. This leads to extended scar formation observed in typical HS skin architectural distortion [80, 89–91]. Matrix remodeling is further amplified by inflammatory signaling driving MMPs activity, generating bioactive matrix fragments that amplify inflammation in a positive loop [77] (Fig. 3).

Immunologically, advanced lesions exhibit prominent TLS formation adjacent to sinus tracts, orchestrated by lymphoid-organizing chemokines such as CXCL13 and CCL19 [67]. Within these structures, B cells differentiate into plasma cells, supporting local antibody production and sustained antigen presentation that perpetuate the already present inflammation rather than promoting resolution [92]. Therefore, Hurley Stage III is characterized by a self-perpetuating microenvironment that maintains fibrosis, chronic inflammation, and tissue destruction in end-stage HS [77, 89, 91] (Fig. 3).

## Inflammation, tissue remodeling and fibrosis in HS pathogenesis: the complex role of MMPs and TIMPs

The structural deconstruction and subsequent fibrotic scarring that define HS are fundamentally governed by a breakdown in ECM homeostasis. This section details the critical, dual-edged roles played by MMPs and their endogenous inhibitors, TIMPs, in driving this pathology. We examine the profound dysregulation of the gelatinases and collagenases MMP-2, −8, and −9, exploring how their hyper-activation transitions from localized tissue destruction—such as hair follicle rupture and sinus tract formation—to systemic inflammatory and metabolic complications. We also evaluate the counter-balancing network of TIMPs (1–4), establishing how a disruption in the delicate MMP:TIMP ratio shifts tissue dynamics from healthy physiological turnover to a self-perpetuating, maladaptive fibrotic epilogue. Together, these sections illuminate how an uncoupled remodeling apparatus transforms an acute inflammatory response into chronic, irreversible architectural distortion.

### Dysregulation of MMP-2, −8, and −9 in HS: from localized matrix remodeling to systemic inflammatory and metabolic perturbations

Key players of inflammation, tissue remodelling and fibrosis in HS pathogenesis are MMPs. MMPs are a family of zinc-dependent endopeptidases that degrade ECM components, while their activity is tightly controlled by their endogenous inhibitors, the TIMPs. MMPs are indispensable for physiological tissue remodeling processes [80]. Their canonical activity is the degradation of ECM proteins, even if they exhibit pleiotropic functions, modulating a diverse array of biological processes, such as cell migration, leukocyte activation and inflammatory responses [89, 92].

In HS, the expression and activity of MMP-2, −8 and −9 are significantly dysregulated [74, 93, 94] (Fig. 3, Table 1). MMP-2, also known as gelatinase A, is a zinc-dependent type IV collagenase [95] secreted as a proenzyme by fibroblasts and endothelial cells [96, 97]. Its substrates include gelatin, elastin, and various other types of collagens [98]. The role of MMP-2 in fibrosis is complex and can be contradictory depending on the tissue and disease stages [99]. For instance, in a rodent model of toxin-induced chronic liver injury, MMP-2 deficient mice demonstrated an almost twofold increase in liver fibrosis compared to wild-type mice [100], due to an enhanced type I collagen mRNA expression. However, the molecular mechanisms underlying the effects of MMP2 deficiency on the up-regulation of type I collagen remain to be deepened. In other cellular contexts, MMP-2 can play pro-fibrotic functions. For instance, in aortic rings and vascular smooth muscle cells of rat models MMP-2 activates TGF-β1 by engaging the SMAD signaling pathway, resulting in the production of the matrix molecules such as fibronectin, and collagen [101].

**Table 1:** Specific Findings on MMP-2, MMP-8, MMP-9 and TIMPs in HS

MMPs	Expression levels	Identified cellular sources	Observed correlations	Mechanisms in HS
MMP-2	Significantly Elevated in Lesional Skin [74, 93, 94]	Keratinocytes, Fibroblasts, Macrophages, Lymphocytes, Sweat Glands, Hair Follicles, Sinus Tracts [95–97]	Positive correlation with Hurley Stages, HSS Score (via C1M, C4M) [104, 105]	Drives expansive lesion growth and sinus tract formation; May inactivate HBD2, impairing antimicrobial defense [93, 100, 101, 103]
MMP-8	Highly Upregulated in Lesions and Blood [102]	Neutrophilic Granulocytes, Dermal Fibroblasts [91, 108]	Positive correlation with TNF-α blood levels, Sartorius Score (inflammatory nodules/abscesses, fistulas); Negative correlation with HDL-cholesterol; Positive correlation with resistin [109]	Contributes to skin cavity (abscess, sinus tract) development; Linked to systemic metabolic alterations [106, 123–125]
MMP-9	High in Lesional Skin (Active Form Present) [84, 110]	Myeloid Cells (Macrophages, Neutrophils), Keratinocytes, Inflammatory Cells, Fibroblasts [112–114]	Positive correlation with Hurley Stages, HSS Score (via C3M, C4M) [104]	Involved in tissue matrix remodeling for lesion/tract development; Part of a causal chain linking HFD, gut dysbiosis, systemic inflammation, HHcy to HS pathology [111, 115, 116, 119]
TIMP-1	Nor reported	Fibroblasts, Mesenchymal Stem Cells, monocytes and macrophages, Endothelial cells, Cancer-Associated Fibroblasts [119, 126–128]	Not reported	High levels contribute to fibrosis by reducing ECM degradation and upregulating inflammatory mediators [89, 116, 129, 130]
TIMP-2, −3, −4	Not reported	Fibroblasts, Mesenchymal Stem Cells, monocytes and macrophages, Endothelial cells, Cancer-Associated Fibroblasts [126, 127, 130, 131]	Positive correlation of TIMP2 with treatment response (HiSCR fulfilment) [124]	Not reported

MMP-2 expression is vigorously elevated in HS lesional skin [74, 93, 94]. Within HS lesions, MMP-2 is detected in keratinocytes, fibroblasts, inflammatory cells (specifically macrophages and lymphocytes), sweat glands, outer epithelial sheath of hair follicles and sinus tracts [93]. Its increased production, directly associated with HS lesional skin, contributes to the formation of hypodermal tracts through extensive matrix remodelling [102] (Fig. 3).

Beyond its role in extracellular matrix remodeling, MMP-2 may exacerbate the chronic inflammatory and infectious nature of HS. Indeed, the excessive expression of MMP-2 and almost absent expression of HBD-2, an anti-microbial peptide, has been found in HS biopsies [93, 103]. Specific ECM-based biomarkers indicative of MMP-2 activity, such as C1M (MMP-2, −9, −13 mediated degradation of type I collagen) and C4M (MMP-2, −9, −12 mediated degradation of type IV collagen), are significantly elevated in higher Hurley stages (II and III) of HS and show a moderate-strong correlation with the Hidradenitis Suppurativa Score (HSS), a classification system of the disease severity, reflecting ongoing dermis degradation and disease activity [104, 105] (Table 1, Fig. 3).

MMP-8, also known as collagenase-2, is a specific collagenase released by polymorphonuclear neutrophils (PMNs) that preferentially cleaves fibrillar collagens, notably types I and III [91]. Being strongly associated with neutrophils distribution, MMP-8 is considered a key mediator in the early inflammatory phase of fibrosis. In murine models of pulmonary and hepatic fibrosis, MMP-8 actively promotes fibrotic responses [106, 107]. MMP-8 is identified as one of the most highly upregulated molecules in HS lesions and blood plasma when compared to healthy controls [102]. A clear positive association exists between MMP-8 and TNF-α blood levels in HS patients. This correlation is mechanistically supported by experimental findings demonstrating that TNF-α stimulates the release of MMP-8 from neutrophilic granulocytes and synovial fibroblasts [108]. The secreted MMP-8, in turn, can activate other pro-inflammatory cytokines, further amplifying the cycle of ECM degradation. In line with this observation, blood MMP-8 levels showed a positive correlation with HS disease severity [109]. As an enzyme specialized in ECM degradation, particularly collagen type I, MMP-8 is hypothesized to contribute directly to the development of abscesses and draining sinus tracts characteristic of HS lesions. Beyond its local effects, a significant association between MMP-8 and metabolic alterations in HS patients has been revealed. Specifically, MMP-8 blood levels negatively correlated with high-density lipoprotein (HDL) cholesterol (an anti-atherogenic lipid) and positively correlated with blood levels of resistin, an adipokine implicated in atherosclerosis and inflammation [109]. The association of MMP-8 with metabolic perturbations suggests a systemic component of HS pathophysiology that extends beyond localized skin inflammation. This observation positions MMP-8 as a potential biomarker not only for skin disease activity but also for systemic comorbidities, offering a broader diagnostic and prognostic utility and potentially linking HS to wider metabolic dysregulation (Table 1, Fig. 3).

Finally, MMP-9, also known as gelatinase B, primarily degrades type IV collagen [110, 111]. It is produced by different cell types including keratinocytes and immune cells, and its activity is enhanced during the early inflammatory stage of wound healing [112–114]. Elevated levels of MMP-9 are frequently observed in a range of fibrotic conditions, including Idiopathic Pulmonary Fibrosis (IPF) and Systemic Sclerosis (SSc) [115–117]. Decreased MMP-9 activity has been reported in the context of hypertrophic scarring, while high levels have been observed in scarless wound healing models [118], leading to the hypothesis that elevated MMP-9 levels might actually contribute to reduced scar formation. MMP-9 expression and secretion are modulated by mechanical cues derived from the ECM. For instance, fibrotic rigidities have been shown to downregulate MMP-9 expression and secretion in hepatic stellate cells involved in liver fibrosis [119]. This observation introduces a feedback loop where the fibrotic environment itself, through increased stiffness, actively suppresses the activity of the enzyme MMP-9, and simultaneously increases the expression of its inhibitor expression TIMP-1, a pro-fibrotic player [120]. This suggests a mechanism by which fibrosis can become self-perpetuating and resistant to resolution, which is vital for understanding the progressive nature of fibrotic diseases. High levels of MMP-9 are present in HS perilesional and lesional skin [84]. Active forms of MMP-9 are detected specifically around sites of active tissue remodeling, including dermal fissures, and are primarily expressed by macrophages and neutrophils [102, 110]. ECM-based biomarkers reflecting MMP-9 activity, such as C3M (MMP-9 mediated degradation of type III collagen) and C4M (MP-2, −9, −12 mediated degradation of type IV collagen), are significantly elevated in Hurley stages II and III of HS and show a moderate-strong correlation with the IHS score, indicating their utility in assessing disease activity and severity [104] (Table 1, Fig. 3).

It has been suggested that MMP-9 upregulation in HS can be a consequence of systemic dysbiosis-led inflammation and hyperhomocysteinemia. Specifically, a high-fat diet in HS patients can initiate gut dysbiosis, often characterized by decreased producers of Short-Chain Fatty Acids, such as *Faecalibacterium prausnitzii* and *Bifidobacterium,* and increased pro-inflammatory bacteria, leading to systemic inflammation [120–122]. This last, coupled with hyperhomocysteinemia, promotes the infiltration of leukocytes into the skin, which release additional inflammatory cytokines that stimulate MMP-9 expression and activity, leading to the formation of painful nodules, and hypodermal tracts [102] (Fig. 3).

### Differential functions of TIMP-1, TIMP-2, TIMP-3, and TIMP-4 in tissue homeostasis and maladaptive remodeling

The function of MMPs is tightly controlled by TIMPs, which form 1:1 complex with MMPs, thereby inhibiting their proteolytic activity of the ECM components [132]. A disruption in this delicate balance between MMPs and TIMPs is a central mechanism contributing to the disturbed collagen deposition and degradation observed in fibrotic conditions [126, 127]. Indeed, the dysregulated production and combined activity of different MMPs and TIMPs, in association to chronical inflammation, can hinder normal ECM turnover and architecture restoration, leading to fibrotic epilogue [84, 123–125].

Elevated levels of TIMP-1 are frequently observed in fibrotic skin conditions, such as Systemic Sclerosis (SSc), age-related renal fibrosis, and cardiac fibrosis and are considered to actively contribute to the development of tissue fibrosis [123, 124] (Table 1). TIMP-2 inhibits most of MMPs, but it also has a dual action on MMP-2. It facilitates the cell-surface activation of pro-MMP-2 by forming a tri-molecular complex with membrane type 1-MMP (MT1-MMP), which is critical in maladaptive cardiovascular ECM remodelling [130]. Furthermore, TIMP-2 can induce a pro-fibrotic response (myofibroblast activation and increased collagen synthesis) in a manner potentially independent of its MMP-inhibitory activity [130] (Table 1). In contrast, TIMP-3 can have anti-fibrotic functions, by inhibiting the enzyme ADAM17 (also known as TNF-α Converting Enzyme, or TACE) [133] (Table 1). This action blocks the cleavage and release of membrane-bound TNF-α, thereby restricting pro-inflammatory and pro-fibrotic signalling [133, 134]. In addition, TIMP-3 can inhibit the pro-fibrotic factors ADAMTS4 and ADAMTS5 (ADAM with Thrombospondin motifs) that, through the cleavage of TGF-β binding proteins, lead to the release of active TGF-β, a potent mediator of fibrosis. By inhibiting ADAMATS members, TIMP-3 can indirectly reduce the amount of active TGF-β, thus mitigating the fibrotic response [101, 135–137] (Table 1). Studies in animal models of kidney injury [138] and pulmonary fibrosis [139] strongly support the anti-fibrotic role for TIMP-3. Finally, like all TIMPs, TIMP-4 inhibits various MMPs, with a notably strong affinity for MMP-26 and the ability to regulate the activation of pro-MMP-2. By inhibiting these enzymes, TIMP-4 helps to regulate the ECM turnover and tissue remodelling in the heart and the brain [134–136] (Table 1). To date, TIMPs role in HS pathogenesis is totally missing. Further in vitro and in vivo studies would be addressed to deepen their expression and functions in the different stages of HS and their specific involvement in inflammation and fibrotic processes.

## Adipose tissue dysfunction in HS

The connection between obesity—the primary comorbidity of HS—and disease pathogenesis extends far beyond simple correlation. Obesity represents a state of chronic, low-grade systemic inflammation, driven by hypertrophic adipose tissue [7, 124]. No longer viewed as merely a passive energy storage site, adipose tissue functions as a highly active, complex endocrine and immunologic organ. While primarily composed of adipocytes, it also contains blood vessels, fibroblasts, and immune cells such as macrophages and T-cells. Beyond standard cytokines, adipose tissue secretes specialized signaling proteins called adipokines, which regulate systemic inflammation, metabolism, and immune responses [124]. In healthy individuals, adipose tissue maintains a homeostatic balance of these proteins. However, in patients with HS- particularly those with elevated body mass indices- this tissue becomes dysfunctional, triggering a severe imbalance between pro- and anti-inflammatory adipokines [84, 125]. Given that adipokines modulate both innate and adaptive immune responses, as well as extracellular matrix (ECM) remodeling and tissue fibrosis, it is evident that adipose tissue plays a pivotal role in driving HS pathogenesis.

### Adipokine signaling in HS: bridging the gap between systemic metabolic dysfunction and cutaneous inflammation

The dysregulated levels of adipokines are not merely markers of obesity but are direct molecular messengers that bridge the gap between systemic metabolic dysfunction and local cutaneous pathology [84, 125]. Indeed, adipokines regulate not only systemic metabolism but also inflammation, immune function, angiogenesis and wound healing [125]. This extensive network of communication allows adipose tissue to integrate systemic metabolism with immune function through both pro- and anti-inflammatory properties. Leptin, resistin, and visfatin are among the key pro-inflammatory adipokines found to be upregulated in obesity and MetS. Leptin regulates appetite and energy expenditure in physiological conditions. However, high levels can cause leptin resistance in the brain, leading to increased appetite and obesity. It also acts as a pro-inflammatory cytokine, influencing both innate and adaptive immune systems and contributing to systemic Th1/Th17 inflammation [125, 140]. Specifically, leptin promotes macrophage polarization towards a pro-inflammatory M1 phenotype, characterized by the production of cytokines such as IL-6, IL-1β, and TNF-α, by activating several signaling pathways, including the mTORC2/AKT, MAPK, and PI3K [141, 142]. In addition, leptin has been demonstrated to enhance immature dendritic cell migration and chemotaxis, promoting a Th1-polarized immune response [143, 144]. Finally, it promotes proliferation and activation of Th1 and Th17 cells, by upregulating glucose metabolism, and suppresses the proliferation of the anti-inflammatory Treg lymphocytes [143, 144].

Resistin directly contributes to insulin resistance in the liver and muscles, hindering glucose uptake [145]. It plays also an important role in the development and progression of chronic type-2 diabetes, hypertension, atherosclerosis, acute myocardial infarction, and heart failure, although the precise mechanisms involved have not yet been fully elucidated [146, 147]. However, resistin is known to promote IL-23/Th17 inflammation in various experimental models and to regulate the homeostasis of Treg cells [125, 148].

The role of visfatin (also known as Nicotinamide Phosphoribosyltransferase or NAMPT) in obesity and MetS is complex. Visfatin has a crucial role in cellular metabolism by contributing to NAD (nicotinamide adenine dinucleotide) biosynthesis and enabling energy metabolism. Following secretion, visfatin also exerts pro-inflammatory activity. Indeed, it induces the synthesis of TNF-α, IL-1β and IL-6 in human leukocytes, as well as in epidermal keratinocytes and fibroblasts, by activation of NF-κB, p38 and MEK1 signaling pathways [149, 150].

In contrast, the role of adiponectin is fundamentally protective. It acts as a key anti-inflammatory and insulin-sensitizing hormone. In individuals with obesity and MetS, the core pathology is a deficiency of adiponectin, which removes its beneficial effects and directly contributes to the development and progression of insulin resistance, dyslipidemia, and cardiovascular disease [151, 152]. Adiponectin signalling suppresses the expansion of antigen-specific T cells and their expression of cytokines, while enhancing T cell apoptosis [153]. In vitro, adiponectin inhibits Th1 cell and Th17 cell differentiation, but not Th2 cell differentiation, through inhibition of RORγt (retinoid-related orphan receptor-γt) [154–156].

**Fig. 4:**
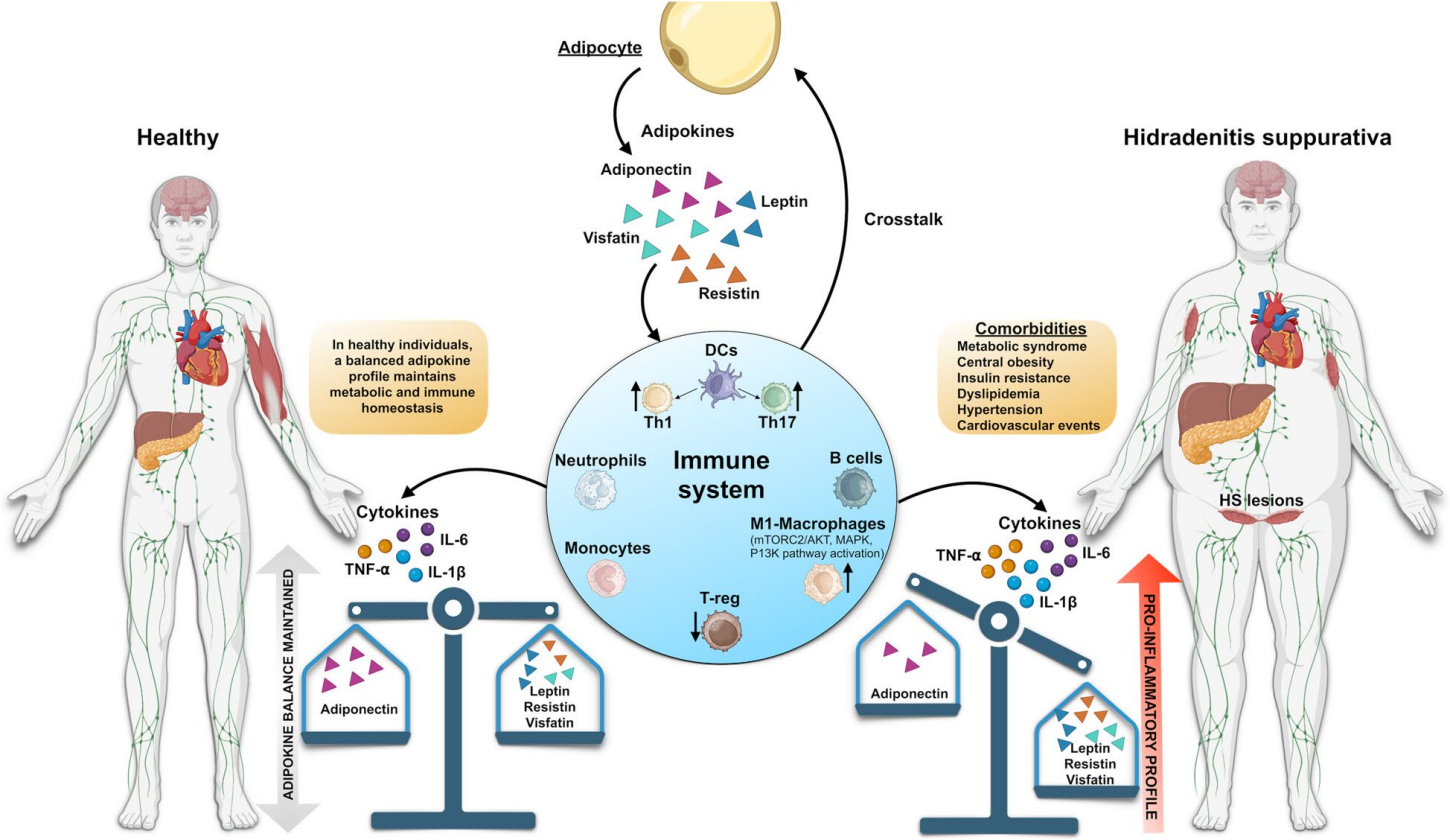
Crosstalk between adipose tissue, immune response and comorbidities in HS. This schematic representation depicts the interaction between adipokines and immune cells in HS compared with healthy tissue. In HS, dysregulated adipokine production—characterized by elevated leptin, resistin, and visfatin and reduced adiponectin—drives a pro-inflammatory cytokine profile (TNF-α, IL-6, IL-1β, IL-17). These changes promote immune cell activation (Th1, Th17, macrophages, dendritic cells) and extracellular matrix remodeling, contributing to skin inflammation and fibrosis, as well as to systemic comorbidities. In healthy individuals, balanced adipokine signaling maintains metabolic and immune homeostasis

In metabolically healthy individuals, a homeostatic balance between these opposing adipokine groups is maintained [157]. However, in patients with HS, this balance is profoundly shifted towards pro-inflammatory adipokines, due, at least in part, to their comorbid condition of obesity [7, 158] (Fig. 4). Indeed, serum concentrations of leptin are notably higher in HS patients than in control individuals and contribute to the state of chronic low-grade inflammation [159]. Resistin and visfatin levels are also significantly elevated and are independently associated with an increased risk for HS [159]. Conversely, adiponectin, a crucial anti-inflammatory adipokine, is found at significantly lower serum concentrations in these patients [160] (Fig. 4).

The upregulation of specific molecules, like leptin, resistin and visfatin, and the downregulation of the adiponectin provide a tangible and mechanistic link that explains how obesity-induced adipose tissue hypertrophy directly drives the inflammatory and metabolic dysregulation observed in HS (Fig. 4). This understanding suggests that therapeutic strategies targeting the adipokine pathways themselves, rather than just focusing on weight loss, may be effective for a broad range of HS patients, regardless of their body mass index (BMI).

### Adipokine-mediated ECM remodelling and fibrosis in HS

**Fig. 5:**
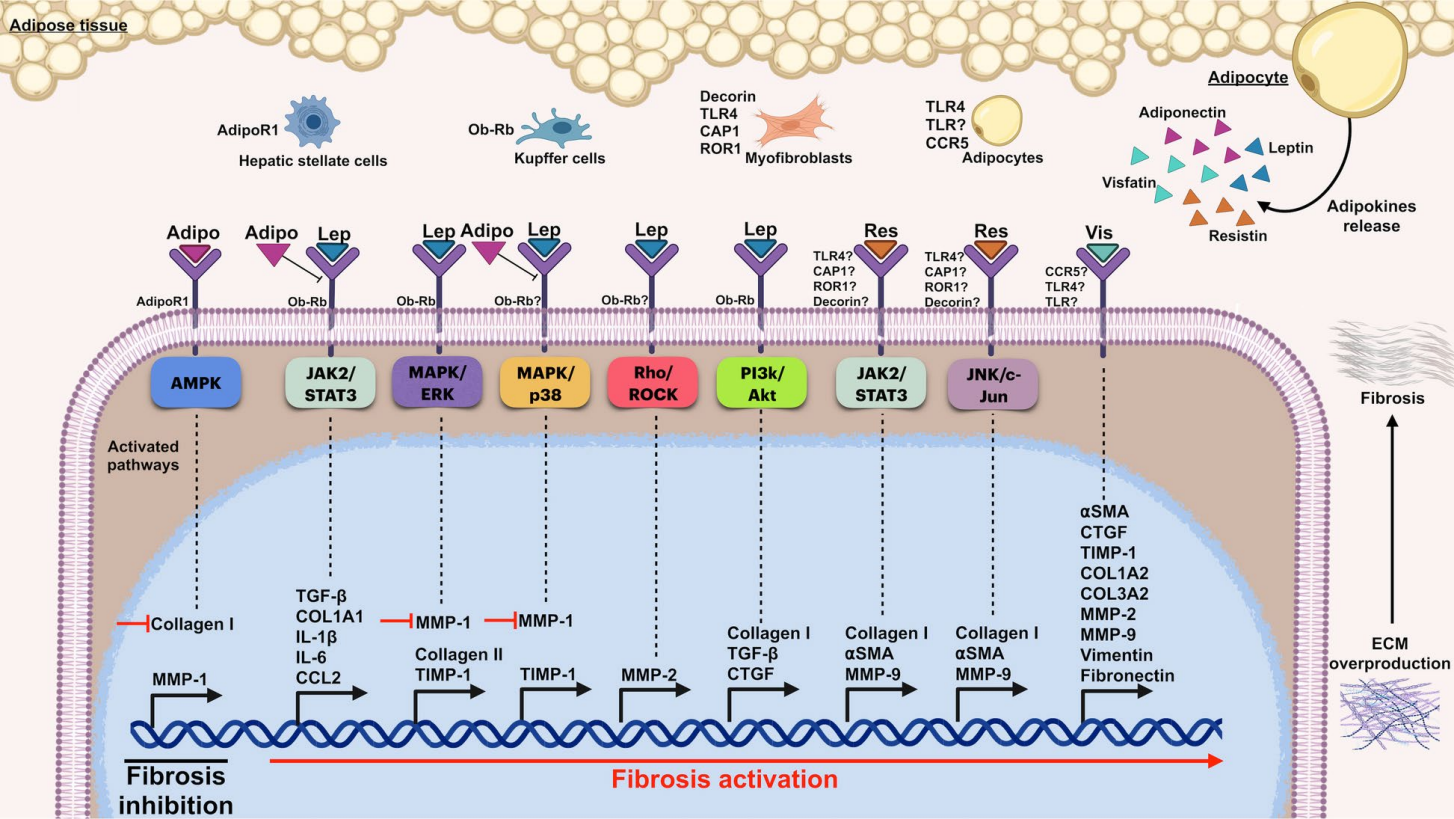
Molecular interplay between leptin, resistin, visfatin, and adiponectin in inflammatory and fibrogenic pathways. Under inflammatory conditions, adipocytes release adipokines such as leptin (Lep), resistin (Res), and visfatin (Vis), which promote fibrogenic signaling. These adipokines downregulate the expression of the antifibrotic gene *MMP1* and upregulate profibrotic genes like TGF-β, COL1 and 2 (collagen type I and II), MMP2, aSMA, resulting in excessive ECM production and increased ECM stiffness that exacerbate tissue fibrosis. Mechanistically, leptin exerts its pro-fibrotic effects through activation of the JAK2/STAT3, MAPK/ERK, p38, Rho/ROCK, and PI3K/AKT pathways, whereas resistin predominantly signals via JAK2/STAT3. Conversely, adiponectin (Adipo) exerts anti-fibrotic activity by inhibiting leptin-induced profibrotic pathways (JAK2/STAT3, MAPK/ERK, and p38) and activating AMPK signaling. Through AMPK activation, adiponectin enhances *MMP1* expression while repressing *COL1* gene expression, thereby counteracting fibrogenesis and maintaining tissue homeostasis

Emerging evidence reveal a tightly interplay between adipokines and ECM remodelling/fibrosis axis. Leptin drives fibrosis starting different intracellular pathways downstream of the long receptor isoform Ob-Rb, including Janus kinase/signal transducer and activator of transcription (JAK)/STAT pathways [161], as well as ERK and AKT-dependent pathways, which activate HSCs proliferation [162]. The activation of JAK2/STAT3 [163] induces the expression of the pro-fibrotic mediators TGF-β1, collagen type I, IL-1β, IL-6, and CCL2 particularly in Kupffer cells and sinusoidal endothelial cells [164, 165] (Fig. 5). In non-alcoholic steato-hepatitis, increased expression of Ob-Rb correlates with the severity of hepatic fibrosis and elevated TGF-β1 level [166], suggesting a crucial role of this pathway. Similarly, in obesity-associated heart failure, adipokine dysregulation promotes cardiovascular fibrosis by driving fibroblast-to-myofibroblast transition and ECM accumulation. In particular, leptin, secreted by epicardial fat and myocardial tissue, contributes to collagen accumulation [167, 168].

In adult rat cardiac myofibroblasts, leptin stimulates expression of collagen I, TGF-β, connective tissue growth factor (CTGF), and galectin-3 through the activation of the PI3K/AKT pathway [169, 170] (Fig. 5). Leptin also enhances ECM accumulation by regulating MMPs/TIMPs system, through the activation of MAPKs such as ERK and p38 pathways that lead to the suppression of collagen-degrading enzymes MMP-1 expression while upregulation of its inhibitor TIMP-1 [171, 172]. In LX-2 hepatic stellate cells, leptin robustly increases TIMP-1 mRNA, protein, and promoter activity through activation of JAK1/2, STAT3/5, ERK1/2, and p38 signaling in an H₂O₂-dependent manner. Pharmacological inhibition of these pathways with specific inhibitors AG490 (JAK inhibitor), PD098059 (ERK inhibitor), SB203580 (p38 inhibitor), or catalase (H₂O₂ scavenger), abrogates this response [171] (Fig. 5). Beyond MMP inhibition, leptin-induced TIMP-1 promotes myocardial fibrosis via CD63/β1-integrin signaling in cardiac fibroblasts [173]. Finally, leptin modulates myocardial matrix remodeling by promoting MMP-2 activation and cardiac fibroblast migration through Rho/ROCK-dependent regulation of MT1-MMP localization [174].

Adiponectin, instead, exerts context-dependent regulatory effects on cardiac and liver fibrosis. It can induce cell migration, MMP activation, and collagen remodeling through the APPL1-AMPK molecular axis [175], while in other settings adiponectin inhibits fibrosis via the AdipoR1-AMPK-iNOS pathway [176]. In an angiotensin-induced cardiac remodeling model, it exerts anti-fibrotic effects through PPAR-α activation [177]. Additionally, adiponectin counteracts leptin effects and restores the proteolytic balance of MMPs/TIMPs system (Fig. 5). In hepatic stellate cells (HSCs) adiponectin inhibits leptin-stimulated JAK2/STAT3 signaling and reduces TIMP-1 expression, through activation of AMP-activated protein kinase (AMPK) and induction of suppressor of cytokine signaling-3 (SOCS-3) [178]. Adiponectin also increases the expression of protein tyrosine phosphatase-1B (PTP1B), which enhances SOCS-3 binding to the leptin receptor Ob-Rb, thereby blocking leptin-mediated formation of extracellular TIMP-1/MMP-1 complex [179], thus resulting in anti-fibrotic effects (Fig. 5).

Conversely, Ramezani-Moghadam and colleagues have demonstrated that adiponectin increases TIMP-1 expression to initiate the MMP-independent signaling cascade through the CD63/β1-integrin complex, leading to the reduction of focal adhesion kinase phosphorylation, thereby limiting HSCs cell migration [180]. They also found a positive association between adiponectin and TIMP-1 serum levels in patients with liver fibrosis [180]. Of interest, in patients with keloids, serum adiponectin are significantly reduced and are inversely correlate with severity index score of disease, while TGF-β1, CTGF, IL-6, and TNF-α levels are elevated compared to controls [181]. Adiponectin also reduces TGF-β1-induced collagen I expression and increases MMP-1 expression in keloid, but not in normal fibroblasts, attenuating fibrotic processes. This inhibition is reversed by the AMPK inhibitor Compound C but unaffected by the PI3K/AKT inhibitor LY294002 [181] (Fig. 5). Adiponectin activates dermal fibroblasts, by inhibiting TGF-β1-induced proliferation and p38-mediated myofibroblast differentiation [182]. Similar effects are observed in keloid fibroblasts, where the adiponectin mimetic ADP355 suppresses ERK and p38 activation, reducing ECM accumulation [183]. In primary human hepatocytes, adiponectin can also increase MMP-9 mRNA and enzymatic activity [184], which plays a bidirectional regulatory role in different fibrotic diseases or different stages of the same fibrotic disease [115]. Due to the lack of mouse models of HS, the bidirectional link between adiponectin and MMP-9 remains to be investigated.

Resistin has emerged as a potent pro-fibrotic mediator, promoting fibroblast-to-myofibroblast trans-differentiation, enhancing the expression levels of type I collagen**,** α-SMA, MMP-9 molecules via JAK2/STAT3 and JNK/c-Jun signaling pathways [185] (Fig. 5). The receptor mediating resistin effects in fibroblast-to-myofibroblast conversion has not yet been identified. Several receptors have been reported to mediate resistin signaling, including adenylyl cyclase-associated protein 1 (CAP1), decorin, receptor tyrosine-like orphan receptor 1 (ROR1), and Toll-like receptor 4 (TLR4), which activate distinct signaling pathways in a tissue-dependent manner. ROR1 and decorin are mainly expressed in pre-adipocytes and adipocytes, respectively, CAP1 in monocytes, while TLR4 is broadly expressed, including in dermal fibroblasts [186, 187].

Finally, visfatin exacerbates hepatic fibrosis in a steato-hepatitis mouse model, where it enhances the expression of fibrosis markers such as αSMA, CTGF, TIMP-1, collagen 1α2, collagen 3α2, fibronectin, and vimentin in liver [188]. Its neutralization by a specific antibody (ALT-100) attenuates disease severity in a murine model of non-alcoholic fatty liver disease, attenuating the progression to hepatic fibrosis [189]. Visfatin is also involved in adipose tissue fibrosis promoting ECM protein production including Collagen type VI, MMP-2 and MMP-9 [190] (Fig. 5). The receptor responsible for mediating these effects has not yet been conclusively identified. Visfatin has been reported to interact with the G-protein-coupled receptor C–C chemokine receptor type 5 (CCR5) [191] and with TLR4 in inflammatory contexts [192]. However, evidence showing that visfatin can exert its effects independently of TLR4 [193] suggests that yet unidentified receptors could be involved in these mechanisms.

Together, these findings outline a causal chain in which obesity-driven systemic adipokine dysregulation activates pro-fibrotic pathways in the skin, although the molecular mechanisms linking adipokines to MMP/TIMP system and extracellular matrix remodeling in HS remain incompletely defined.

## Potential implications of HS treatments on inflammation and tissue architecture

### Pharmacological modulation of HS: from conventional antibiotics to precision biologic interventions

The management of moderate-to-severe HS increasingly relies on conventional and targeted therapies, which interrupt broad or specific inflammatory pathways to reduce disease activity, respectively.

**Fig. 6:**
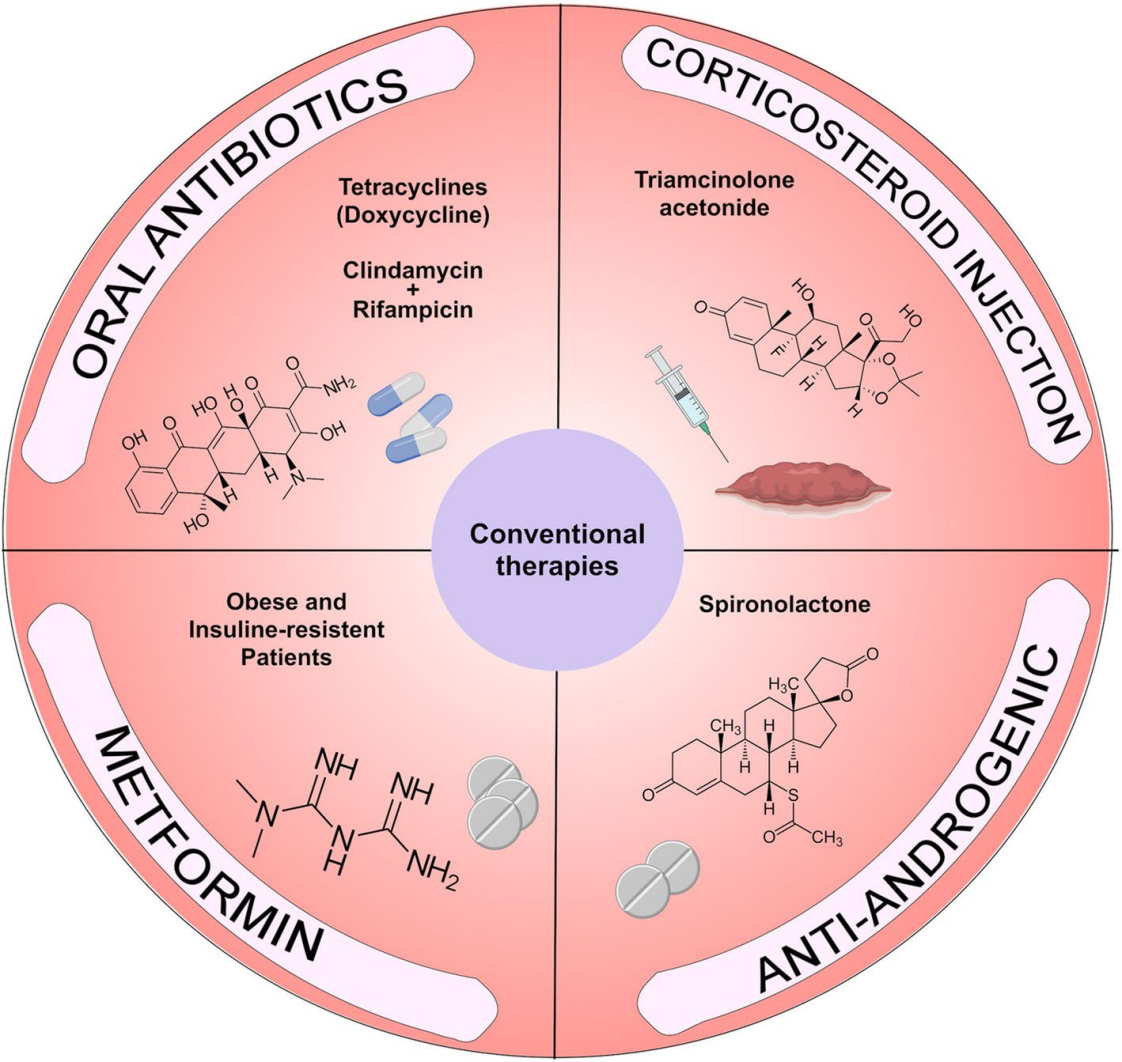
Categories of conventional therapies for HS management. The Fig. provides an overview of conventional therapeutic strategies for patients with HS. First-line and advanced conventional treatments depicted include oral antibiotics (tetracyclines such as doxycycline, or combination of clindamycin and rifampicin), intralesional corticosteroid injections (triamcinolone acetonide), and anti-androgenic therapy (spironolactone). Additionally, metformin has emerged as an adjunctive option in obese and insulin-resistant patients affected by HS

Conventional therapies include oral antibiotics, specifically tetracyclines (in particular doxycycline), considered first-line systemic agents for mild to severe HS forms [8], anti-androgenic therapies (specifically spironolactone), particularly relevant in female patients [194], and intralesional corticosteroid injections (i.e. triamcinolone acetonide) prompting reductions in pain and inflammation [195, 196]. Doxycycline is effective primarily for mild-to-moderate cases, with a 54% reduction in Sartorius score [197]. Patients with more advanced disease, characterized by sinus tracts and scarring, often require a combination of antibiotics, such as clindamycin and rifampicin [198, 199] (Fig. 6). Approximately 48.2% of patients achieve a 50% or greater reduction in inflammatory lesion counts after a 12-week course with the combination of Clindamycin and Rifampicin [200].

In addition, acitretin, a derivative of vitamin A, may be considered in patients with refractory disease who are not of childbearing potential. Overall, around 47% reach a HSS ≥ 50% reduction from baseline [201]. Of note, obesity may reduce treatments efficacy due to altered drug pharmacokinetics and increased systemic low-grade inflammation. Indeed, BMI is associated with a poor response to standard antibiotic courses, requiring careful dosage management to avoid therapeutic failure [202].

Mechanistically, antibiotics are known to inhibit MMPs, as well as to reduce neutrophil chemotaxis and downregulate pro-inflammatory cytokines such as CXCL-8, TNF-α, and IL-1β [201–205]. Corticosteroids, other than inhibiting inflammation, decrease the production of Type I and Type III collagen and glycosaminoglycans, which are the building blocks of HS scars, and interfere with TGF-β signaling, which is the primary driver of the transition from chronic inflammation to permanent scarring [206].

**Fig. 7:**
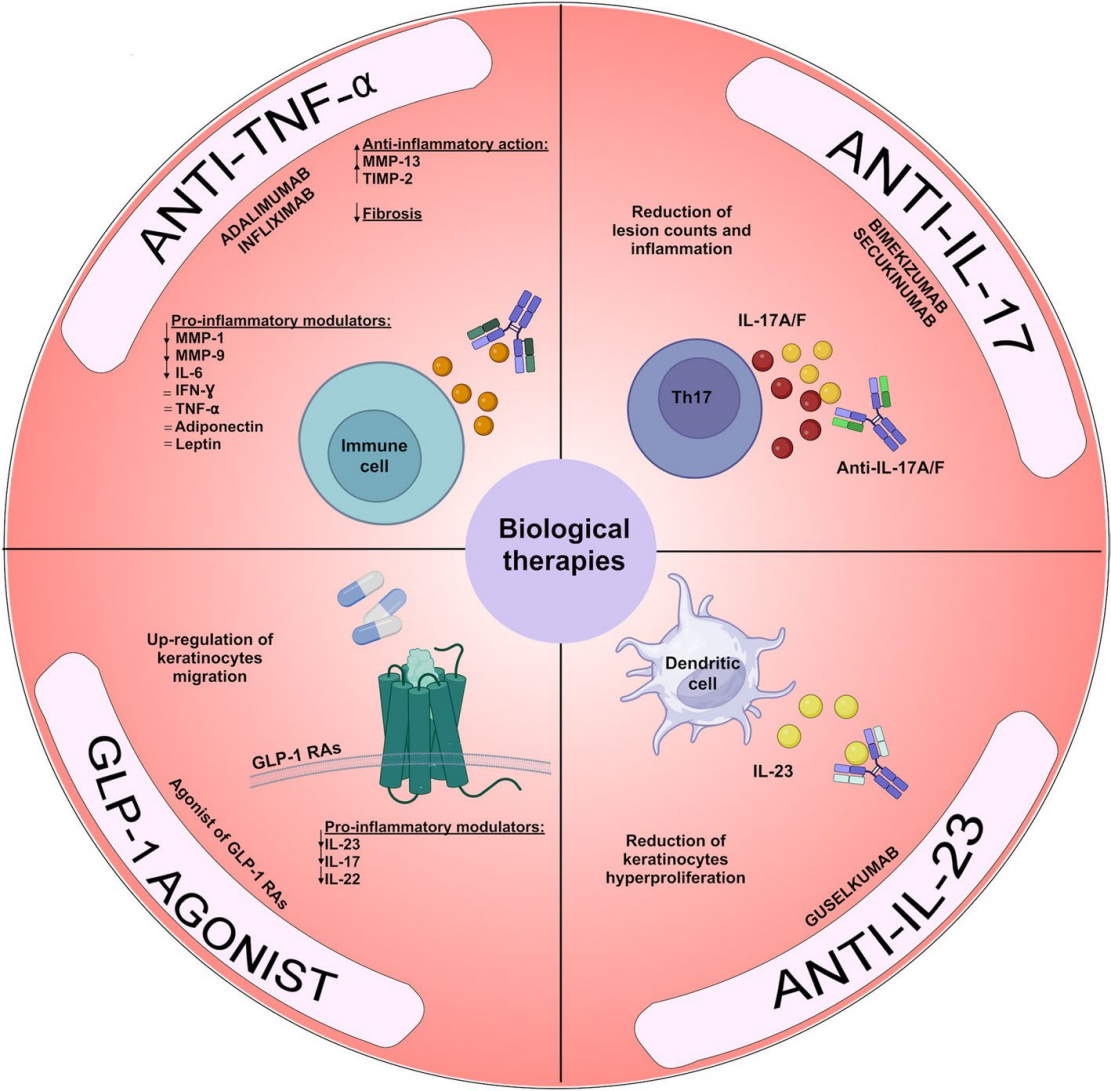
Categories of biologic therapies for HS management. Current and emerging therapeutic approaches targeting inflammatory and fibrotic pathways in HS can be grouped into four categories based on their mechanisms of action. Biologic agents targeting TNF-α signaling (Adalimumab and Infliximab) determines the reduction of pro-inflammatory modulators MMP-1, MMP-9, IL6 and the up-regulation of anti-inflammatory modulators MMP-13 and TIMP-2 resulting in the reduction of fibrotic processes. Antibodies targeting IL-17A/F and IL-23 cytokines (Secukinumab, Bimekizumab, and Guselkumab, respectively) reduce keratinocyte hyperproliferation and neutrophil recruitment. GLP-1 receptor agonists represent a novel class addressing the metabolic-inflammatory axis, combining systemic metabolic improvement with direct anti-inflammatory effects

Of note, since obesity is often linked to insulin resistance, metformin has emerged as an adjunctive option, especially in HS patients, although designed and controlled studies are needed to further evaluate the long-term efficacy of metformin in HS patients [207]. Its potential benefit may derive from metabolic modulation, anti-androgenic effects, and weight reduction [208–210]. To this regard, the emerging use of Glucagon-Like Peptide-1 (GLP-1) Receptor Agonists (GLP-1 RAs) in HS is a compelling area of research, particularly because it targets the metabolic-inflammatory axis that drives the disease [37, 211, 212]. GLP-1 RAs offer a dual mechanism of action through profound metabolic improvement and direct anti-inflammatory effects. Specifically, GLP-1 RAs mimic the GLP-1 hormone [213] whose agonists inhibit IL-23, IL-17, and IL-22, key drivers of psoriasis and HS development [214], as well as upregulate keratinocyte migration by activating P13K/AKT pathway, which is pertinent to wound healing [215] (Fig. 7). GLP-1 RAs also sustain weight loss, leading to less friction and maceration in intertriginous areas, where HS flares are common [29, 216–219]. In obese patients submaximal-dose GLP-1 RA therapy is associated with significant improvements in metabolic parameters and adipokine regulation, but do not affect systemic inflammatory markers within 12 weeks [220].

As an alternative to these treatments, targeted therapies aim to halt the destructive inflammatory cycle involved in molecular pathogenesis and permanent tissue damages characteristic of HS by blocking key immune pathways. Biologic therapies such as Adalimumab (the first FDA-approved biologic for HS) [221–223], and Infliximab bind to and block the activity of TNF-α, a major pro-inflammatory cytokine highly upregulated in HS lesions [224] (Fig. 7). By significantly controlling acute and chronic inflammation, Adalimumab and Infliximab prevents the development of new, irreversible fibrotic processes (new sinus tracts, bridge scars) [225]. Long-term follow-up studies confirmed sustained efficacy, which correlates with long-term prevention of disease progression and severe scarring (Hurley Stage III disease). Specifically, in a meta-analysis study carried by Shih et al. a pooled response rate of 83% HS patients was reported for Infliximab treatment. In PIONEERI and PIONEERII clinical trials for Adalimumab, 41.8% and 58.9% of HS patients reached, respectively, a HiSCR (Hidradenitis Suppurativa Clinical Response), defined as a 50% reduction in the total abscess and inflammatory nodule (AN) count [226]. In this real‐world setting, Adalimumab treatment of moderate‐to‐severe HS resulted in decreased disease severity in 70.2% of HS patients [227]. Finally, in open-label extension studies, the HiSCR was maintained in approximately 52.3% of patients through 168 weeks of continuous weekly treatment [228].

However, a real-world study identified significantly lower efficacy of Adalimumab in HS patients with BMI ≥ 30 compared to those with BMI < 30 [229], suggesting that therapeutic efficacy is inversely correlated to obesity. A recent study of RNA seq carried out on HS patients demonstrated that Adalimumab reduces the levels of inflammatory MMP-1 and MMP-9 while promoting wound-healing MMP-13 and TIMP-2 levels in the circulation of the patients with who responded to treatment [230]. However, in a recent study carried out on patients with HS and control subjects after at least 12 weeks of treatment with Adalimumab, only plasma IL-6 and high-sensitivity C-reactive protein (hsCRP) are reduced by biologic therapy, while IFN-γ, TNF-α, adiponectin and leptin levels do not change [231] (Fig. 7).

Newer biologic agents targeting other crucial cytokines, such as IL-17, have been recently approved for HS treatment. Secukinumab and Bimekizumab work to reduce lesion counts and inflammation by blocking IL-17A and/or IL-17F, which drive keratinocytes hyperproliferation, the neutrophils recruitment, and chronic inflammation [232–234] (Fig. 7). Real-world data on the long-term effectiveness of the anti-IL17 agent Secukinumab in treating moderate-to-severe HS showed a 56.5% maximal response rate (evaluated in terms of HiSCR and IHS4 reduction of at least 50%) at 6 months and dropout exceeding 40% at 1 year of treatment [235]. Similarly, a real-world study showed that 66.67% of HS patients responded to Bimekizumab at 16 weeks as calculated by HiSCR50 [236]. Response rates in this group at week 16 are higher than those seen in phase III clinical trials, where at week 12, 46% of Bimekizumab-treated participants achieved HiSCR75 and 32% achieved HiSCR90 [237].

However, molecular data on the effects of these biologics on inflammation, ECM remodelling or fibrosis pathways are missing. It is plausible that anti-IL-17 biologic affect these processes, as it has been reported that IL-17A and IL-17E contribute to IPF, primary biliary cirrhosis, and salivary gland fibrosis through the activation of epithelial-mesenchymal transition (EMT), which leads to fibroblast proliferation and differentiation in myofibroblasts [238]. Furthermore, case reports or case series also show good results for other biologics. For instance, Guselkumab, an anti-IL-23 biologic, may offer clinical benefit in select HS populations, particularly those with comorbid psoriasis or Crohn's disease [239], although its impact on tissue destruction and fibrosis has not been yet investigated (Fig. 7). However, IL-23, through several signaling pathways, provokes the activation of EMT mechanism, inducing fibrotic processes in autoimmune diseases, and chronic inflammation that determines severe fibrotic evolution in several diseases [239].

Finally, despite substantial advances in medical therapy, surgery remains the definitive treatment for irreversible structural sequelae of HS, including epithelialized sinus tracts and hypertrophic scarring. For localized disease, particularly Hurley stage I and II, the minimal-invasive tissue-saving surgical technique, known as deroofing, is used [240]. Patients with extensive, coalescing disease, typical of Hurley stage III, often require wide radical excision, which entails removal of all apocrine-bearing tissue [241]. However, as for the other standard therapies, higher BMI can sometimes complicate post-surgical wound healing in skin folds due to increased tension on the stitches and moisture in the area.

### Clinical trials for HS treatment: next-generation antibodies and intracellular signaling inhibitors

Beyond currently approved biologics used as standard therapies in HS as well as conventional and surgical treatments, a growing number of clinical trials are evaluating novel therapeutic strategies, based on the refined immune-pathogenesis understanding and the urgent need to better manage patients with refractory disease and extensive structural damages.

Although the IL-17 axis still remains of therapeutic interest, increasing evidence points to JAK signaling and emerging adaptive B-cell involvement in chronic tunnels as molecular targets [3, 242]. Following the regulatory approval of anti-IL17 biologics Secukinumab and Bimekizumab, therapeutic development in HS has increasingly focused on improving their therapeutic efficacy and drug penetration, particularly within fibrotic, tunnel-rich lesions.

Conventional monoclonal antibodies may have limited penetration into the complex HS tunnel microenvironment [233, 243]. Izokibep, a small IL-17A–targeting protein, may improve tunnel penetration and is currently in phase 3 trials following favorable phase 2b results in anti–TNF-α–refractory moderate-to-severe HS [NCT05905783 [242];]. Similarly, Sonelokimab, a trivalent IL-17A/F nanobody with extended half-life by albumin binding, is under phase 3 evaluation with promising efficacy and safety [NCT06411899; NCT06411379]. In parallel, the bispecific IL-17A/F inhibitor Brivekimig is being assessed in phase 2b trials stratified by draining tunnel burden to address phenotypic and fibrotic heterogeneity [NCT07170917] (Table 2).

**Table 2:** Ongoing clinical trials in HS

Drug	Target/mechanism	Drug class	Clinical phase	Key population/rationale	Clinical trial reference
Izokibep	IL-17A	Small protein IL-17A inhibitor	Phase 2b/Phase 3	Moderate-to-severe HS; prior anti-TNF-α failure; improved penetration into fibrotic tunnels	NCT05355805 (Phase 2b); NCT05905783 (Phase 3)
Sonelokimab	IL-17A/IL-17F + albumin binding	Trivalent nanobody	Phase 3	Moderate-to-severe HS; dual IL-17 blockade with prolonged systemic half-life	NCT06411899; NCT06411379
Brivekimig	IL-17A/IL-17F	Bispecific monoclonal antibody	Phase 2b	Moderate-to-severe HS; Enrollment stratified by draining tunnel burden to address phenotypic heterogeneity	NCT07170917
Povorcitinib	JAK1	Selective JAK1 inhibitor (oral)	Phase 2/Phase 3	Moderate-to-severe HS; Clinically meaningful efficacy on inflammatory lesions, pain, and fibrotic remodeling	NCT03569371 (Phase 2); NCT05620823 (Phase 3)
Upatacitinib	JAK1	Selective JAK1 inhibitor (oral)	Phase 3	Moderate-to-severe HS; Assessment of disease activity and safety	NCT05889182
Ruxolitinib cream (1.5%)	JAK1/JAK2	Topical JAK inhibitor	Phase 3	Hurley stage I–II HS; topical modulation of JAK/STAT signaling	NCT06959225
Remibrutinib	Bruton’s tyrosine kinase (BTK)	Selective BTK inhibitor (oral)	Phase 2/Phase 3	Moderate-to-severe HS; Targeting B-cell–driven inflammation	NCT06840392
Fostamatinib	Spleen tyrosine kinase (SYK)	SYK inhibitor (oral)	Open-label/Phase 2	Moderate-to-severe HS; Targeting B-cell populations and downregulation of inflammatory and fibrotic gene signatures in HS fibroblasts	NCT05040698

Beyond blocking cytokines, targeting intracellular molecular pathways implicated in HS pathogenesis have become a promising strategy for HS treatment. In particular, the JAK/STAT molecular pathway has emerged as a key target in the evolving therapeutic landscape for HS. Povorcitinib, a selective JAK1 inhibitor, demonstrated clinically meaningful efficacy in phase 2 studies, prompting advancement into ongoing phase 3 trials evaluating long-term effects on lesion resolution, pain and fibrotic remodeling [244], [NCT03569371, NCT05620823]. Upadacitinib (JAK1 inhibitor) under phase 3 evaluation [NCT05889182] has shown promising results, specifically targeting patients with prior TNF-α inhibitor failure. Topical modulation of JAK signaling is being explored through a 1.5% Ruxolitinib cream formulation, now in phase 3 for patients with Hurley stage I and II disease [NCT06959225]. JAK inhibitors markedly reduce inflammatory lesions and abscesses by broadly suppressing pro-inflammatory cytokine signaling, including key fibrogenic pathways such as IL-6 and IL-17, thereby limiting inflammation-driven fibrosis. Furthermore, as previously described, the JAK/STAT pathway directly contributes to fibrotic mechanisms by upregulating the expression of profibrotic mediators [164, 165], also through the activation by the pro-inflammatory adipokine leptin [161].

These findings suggest that targeting the JAK/STAT axis could provide a dual benefit in HS by attenuating both inflammation and fibrosis, while also addressing the contribution of adipose tissue-derived mediators to these pathogenic mechanisms (Table 2).

In addition, recent advances have focused attention on B cells within chronic HS tunnels, redirecting therapeutic interest toward humoral immune pathways [245]. In this context, the inhibition of Bruton’s tyrosine kinase (BTK), cytoplasmic kinase involved in the development, signaling and survival of B-lymphocytes [246] represents a novel mechanistic approach. The selective oral BTK inhibitor Remibrutinib in phase 2, promoting progression to phase 3 to assess long term efficacy, supports the clinical relevance of the B-cell axis in HS [NCT06840392]. Similarly, inhibition of spleen tyrosine kinase (SYK), with Fostamatinib, an important mediator of downstream signaling in B cells but also involved in modulation of fibroblasts inflammation and fibrotic programs [247], has demonstrated encouraging efficacy in open-label studies [NCT05040698; [248, 249]] (Table 2). Notably, SYK treatment down-regulates the expression of genes related to inflammation, proliferation, fibrosis and migration in fibroblast subpopulations implicated in scarring. Importantly, the inhibition of BTK and SYK pathways, implicated in the amplification NF-κB and MAPK-mediated inflammatory cascades and intersecting with the JAK/STAT network [246, 250], could support a translational link between B-cell-driven inflammation and attenuation of fibrosis in HS lesions.

In conclusion, the HS clinical trial landscape has expanded to diverse therapeutic strategies targeting multiple disease aspects, including fibrosis, scarring, and chronic tunnels. However, variable and limited responses highlight the cellular and molecular heterogeneity of HS, suggesting distinct inflammatory and fibrotic endotypes and limiting the effectiveness of a one-size-fits-all approach. In addition, obesity has emerged as a key disease modifier affecting patient stratification, trial endpoints, and outcome interpretation. Future therapies must integrate patient-specific factors and fibrotic disease features into trial design and treatment selection to achieve meaningful outcomes.

## Discussion and future directions

The prevailing evidence confirms that HS transcends the traditional definition of follicular occlusion. It is a progressive, multi-systemic inflammatory disorder characterized by an aberrant ECM remodeling–fibrosis axis. Central to its pathogenesis is a dysregulated adipokine profile, which acts as a molecular bridge between obesity-related systemic inflammation and localized cutaneous destruction. In this landscape, the imbalance between MMPs and TIMPs -fuelled in part by visceral and subcutaneous adiposity- serves as both a consequence of and a fuel for the chronic inflammatory state.

Despite these insights, clinical management remains suboptimal, even in advanced healthcare systems. The profound impact on patient quality of life is exacerbated by a diagnostic latency of 7–10 years and a "therapeutic lag," where biologics are often introduced only after years of less effective systemic treatments [251]. Moreover, the 40–50% non-response rate to current biologics underscores the influence of the complex genetic profiles on the clinical outcomes. Bridging this gap requires the identification of robust genetic and proteomic biomarkers to facilitate not only the early differential diagnosis but also to predict individual therapeutic response. To this regard, a recent pharmacogenomic GWAS study carried out on 455 HS patients, investigating genetic variants associated with response to Adalimumab, has identified a SNP in the gene of the anti-apoptotic factor *BCL2,* thus suggesting a potential role of apoptosis regulation in the pathophysiology of Adalimumab response. Of note, this study provided the first evidence of a genetic link to Adalimumab response [252].

Addressing the "great unknowns" of HS require a deeper investigation into the functional duality of MMPs and TIMPs. It remains unclear which specific isoforms drive pathology versus those that facilitate repair; their roles likely shift dynamically across disease stages. While broad-spectrum inhibitors (e.g., Batimastat) failed due to dose-limiting musculoskeletal toxicities, highly selective agents like Andecaliximab (targeting MMP-9) offer a more precise alternative. Andecaliximab is a highly selective monoclonal antibody designed to inhibit MMP-9. It represents the most advanced attempt to target this specific protein. While it was studied in Phase II and III trials for Ulcerative Colitis and Gastric Cancer, it failed to meet its primary endpoints in those areas [253, 254]. In 2023, research highlighted that "active" MMP-9 (specifically a form called F107-MMP9) is highly localized in the dermal fissures and fistulae of HS patients. This has sparked renewed interest in using specific antibodies like Andecaliximab for HS, but a formal HS-specific trial has not yet been launched.

Future research should also integrate the influence of the "gut-skin axis" and metabolic factors on HS pathogenesis and the optimal response to drugs. Investigating how diet and the microbiome modulate MMP activity could pave the way for lifestyle-based adjunctive therapies, with emerging evidence suggesting that Mediterranean diet adherence and weight management may significantly improve clinical outcomes.

Ultimately, the transition toward precision medicine in HS necessitates a departure from "single-target" paradigms. We should embrace a network biology framework that accounts for the interplay between inflammatory mediators, adipokines, and specialized fibroblast subtypes. A "multi-hit" therapeutic strategy -simultaneously neutralizing inflammatory "alarmins" (i.e. TNF-α, IL-1β) while stabilizing the ECM remodelling/fibrosis balance and addressing metabolic dysfunction- may be required to achieve durable, long-term remission. Advancing this frontier will depend on large-scale clinical trials that integrate high-resolution "omics" (genomic, transcriptomic, and proteomic) to define distinct disease endotypes and deliver truly personalized care.

To conclude, the management of HS should also address the profound psychological burden that characterizes the disease. The chronic pain, malodour, and visibility of HS lesions often lead to social isolation, anxiety, and depression, creating a bidirectional cycle were psychological distress fuels systemic inflammation and vice versa. Addressing the profound psychological burden is crucial to interrupting the bidirectional cycle between mental health issues and systemic inflammation [4]. Indeed, mental resilience and patient self-efficacy are essential prerequisites for sustaining radical lifestyle changes, such as smoking cessation and dietary adherence, which are necessary to reduce the adipokine-mediated inflammatory burden. Therefore, by acknowledging the unique psychological profile and social circumstances of each patient, clinicians can move beyond standard protocols to develop targeted interventions that improve not only the skin's appearance but the patient's entire quality of life.

## Data Availability

Not applicable.
